# Protective effects of *Mentha piperita* L. leaf essential oil against CCl_4_ induced hepatic oxidative damage and renal failure in rats

**DOI:** 10.1186/s12944-017-0645-9

**Published:** 2018-01-09

**Authors:** Khaled Bellassoued, Anis Ben Hsouna, Khaled Athmouni, Jos van Pelt, Fatma Makni Ayadi, Tarek Rebai, Abdelfattah Elfeki

**Affiliations:** 1 Department of Life Sciences, Animal Ecophysiology Laboratory, Faculty of sciences, University of Sfax Tunisia, Road of Soukra Km 3.5, BP 1171, PC 3000 Sfax, Tunisia; 2 Biotechnology and Plant Improvement Laboratory, Centre of Biotechnology of Sfax, PO Box 1177, Road Sidi Mansour km 6, 3018 Sfax, Tunisia; 3 Department of life sciences, Laboratory of Biodiversity and Aquatic Ecosystems, Faculty of sciences, University of Sfax Tunisia, Road of Soukra Km 3.5, BP 1171, PC 3000 Sfax, Tunisia; 4 Laboratory of Clinical Digestive Oncology, Department of Oncology, KU, Leuven, Belgium; 5 Biochemistry Laboratory, CHU Habib Bourguiba of Sfax, Faculty of Medicine, University of Sfax Tunisia, Road Menzel Chaker km 0.5, CP 3029 Sfax Sfax, Tunisia; 6 Laboratory of Histology and Embryology, Faculty of Medicine of Sfax, University of Sfax Tunisia, Road Menzel Chaker km 0.5, CP 3029 Sfax, Tunisia

**Keywords:** *M. piperita*, Essential oil, Liver, Kidney, Oxidative stress, Histopathological

## Abstract

**Background:**

*Mentha piperita* L. is a flowering plant belonging to the Lamiaceae family. *Mentha* plants constitute one of the main valuable sources of essential oil used in foods and for medicinal purposes.

**Methods:**

The present study aimed to investigate the composition and in vitro antioxidant activity of *Mentha piperita* leaf essential oil (MpEO). A single dose of CCl_4_ was used to induce oxidative stress in rats, which was demonstrated by a significant rise of serum enzyme markers. MpEO was administrated for 7 consecutive days (5, 15, 40 mg/kg body weight) to *Wistar* rats prior to CCl_4_ treatment and the effects on serum alanine aminotransferase (ALT), aspartate aminotransferase (AST), alkaline phosphatase (ALP), lactate dehydrogenase (LDH), and γ -glutamyl transpeptidase (γ-GT) levels, as well as the liver and kidney superoxide dismutase (SOD), catalase (CAT) and glutathione peroxidase (GPx) activity and thiobarbituric acid reactive substances (TBARS) levels were evaluated. In addition, histopathological examinations of livers and kidneys was performed.

**Results:**

The in vitro antioxidant activity of MpEO was lower than that of silymarin. Pretreatment of animals with MpEO at a dose of 5 mg/kg did not have a significant effect on ALT, AST, ALP, LDH, γGT, urea or creatinine levels in CCl_4_-induced stress. Whereas pretreatment with MpEO at doses of 15 and 40 mg/kg prior to CCl_4_, significantly reduced stress parameters (ALT, AST, ALP, LDH, γGT, urea and creatinine) compared to the CCl_4_-only group. Moreover, a significant reduction in hepatic and kidney lipid peroxidation (TBARS) and an increase in antioxidant enzymes SOD, CAT and GPx was also observed after treatment with MpEO (40 mg/kg) compared to CCl_4_-treated rats. Furthermore, pretreatment with MpEO at 40 mg/kg can also markedly ameliorate the histopathological hepatic and kidney lesions induced by administration of CCl_4_.

**Conclusions:**

We could demonstrate with this study that MpEO protects liver and kidney from CCl_4_-induced oxidative stress and thus substantiate the beneficial effects attributed traditionally to this plant.

## Background

Reactive oxygen species (ROSs) are various forms of activated oxygen. A disproportion of the reactive oxygen species and the absence of their scavenge systems in cells lead to oxidative stress and increases the risk of several human chronic diseases [1]. ROS contributes to the development of various diseases such as diabetes, atherosclerosis, cancer, neurodegenerative diseases, liver cirrhosis and the aging process [2]. The liver plays a central role in the maintenance of systemic lipid homeostasis and is especially susceptible to ROS damage. CCl_4_ is now of greatest concern as an environmental contaminant [3]. It was reported that CCl_4_ was one of the most commonly used toxins in the experimental study of liver diseases [4]. Abraham et al. [5] showed that the nephrotoxic effects of CCl_4_ were also associated with free radical production.

To prevent the damage caused by ROS, living organisms have developed an antioxidant defense system that includes the presence of non-enzymatic antioxidants and enzymes such as catalase (CAT), superoxide dismutase (SOD) and glutathione peroxidase (GPx) [6]. It has been anticipated that in addition to these natural antioxidants, other synthetic or natural ROS scavengers may reduce the incidence of free radical-mediated diseases. The use of antioxidants in the prevention and cure of various diseases is intensifying, and there is considerable interest in the study of the antioxidant activities of molecules such as plant polyphenolic and carotenoid components [6, 7]. Antioxidants appear to act against disease processes by increasing the levels of endogenous antioxidant enzymes and decreasing lipid peroxidation [8].

A number of studies showed that various herbal extracts could protect liver and kidney against CCl_4_-induced oxidative stress by inhibiting lipid peroxidation and enhancing antioxidant enzyme activity [9]. Silymarin, a flavonolignan mixture of milk thistle (*Silybum marianum*), is one such important herbal hepatoprotective drug. Silymarin exhibits hepatoprotective effects by altering cytoplasmic membrane architecture and, in turn, preventing the penetration of hepatotoxic substances, such as carbon tetrachloride (CCl_4_), thioacetamide and D-galactosamine [10].

The well-known and widely used peppermint (*Mentha piperita* L.) (Lamiaceae) is a cultivated natural hybrid of *Mentha aquatica* L. (water mint) and *Mentha spicata* L. (spearmint). Although a native genus of the Mediterranean region, it is cultivated all over the world for its use in flavor, fragrance, medicinal, and pharmaceutical applications. Peppermint oil is one of the most widely produced and consumed essential oils [11, 12]. Besides its uses in food, herbal tea preparations, and confectioneries, the medicinal uses of mint, which date back to ancient times, include carminative, anti-inflammatory, antispasmodic, antiemetic, diaphoretic, analgesic, stimulant, emmenagogue, and anticatharrhal application. It is also used against nausea, bronchitis, flatulence, anorexia, ulcerative colitis, and liver complaints. Mint essential oils are generally used externally for antipruritic, astringent, rubefacient, antiseptic, and antimicrobial purposes, and for treating neuralgia, myalgia, headaches, and migraines [13, 14].

From the experimental and clinical studies performed on *Mentha piperita* leaf essential oil (MpEO), it seems that most of its pharmacological actions are due to its antioxidant activity which is mainly due to its ability to scavenge free radicals and/or inhibit lipid peroxidation [15, 16]. Antioxidants are substances that delay or prevent the oxidation of inter- or intra-cellular oxidizable substrates from oxidative stress. In this study, we report the chemical composition and antioxidant effects of MpEO in several in vitro systems (DPPH and superoxide scavenging activities). Besides, we are interested in determining the possible protective effects of MpEO against oxidative damage of the liver and kidney following an intraperitoneal administration of CCl_4_, by assessing the oxidative stress profile and some serum biochemical parameters.

## Methods

### Plant material

Fresh leaves of *M. piperita* L. samples were harvested from the local market at Sfax (Tunisia) (N: 34.4426°, E: 10.4537°) during the vegetative stage in June 2013. The samples were identified and authenticated by a senior botanist, Pr. Ferjani Ben Abdallah, at the Faculty of Science of Sfax, University of Sfax (Tunisia). From 50 individual *M. piperita* L. plants each, a total of 80–100 leaves (≈ 12 cm^2^ in size) were randomly collected from the base to the apex. The fresh leaves were mixed and immediately dried in the shade away from light at room temperature. After drying, the samples were grounded to a fine powder that was used for the extraction of essential oil.

### Essential oil preparation

MpEO was extracted by the steam distillation method. A mass of 3 kg of dry plant material was hydrodistillated for 2 h in a Clevenger-type apparatus. The recovered (0.47%) essential oil was dried with anhydrous Na_2_SO_4_, and stored at 4 °C.

### *Mentha piperita* essential oil composition

MpEO compositional analysis of the volatile constituents was performed on a Hewlett-Packard gas chromatograph GC: 5890 series II. The fused HP-Innowax capillary column (polyethylene glycol, 30 m, 0.25 μm, ID, 0.25 mm film thickness) was directly connected to the mass spectrometer. Nitrogen was used as a carrier gas at a flow rate of 1.2 ml/min. Oven temperature was initially set at 50 °C (1 min) and gradually raised to 250 °C (5 min) at 7 °C/min. The temperatures of the injection port and detector were maintained at 250 and 280 °C, respectively. The mass spectrometer was operated (full scan-mode) in the EI-mode at 70 eV.

### Component identification

The essential oil components were identified based on their mass spectra and computer matching with the data available in the Wiley 275 library (Wiley, New York).

### In vitro antioxidant activities test

The antioxidant activity of the MpEO was determined by two methods and compared with the activity of silymarin, a standardized extract of the milk thistle seeds that containes a mixture of flavonolignans. Silymarin has a number of potential mechanisms including chemoprotective effects from environmental toxins and anti-inflammatory activity and is used as a drug.

### Measurement of free radical-scavenging action

2,2-Diphenyl picrylhydrazyl (DPPH) free radicals scavenging activity was assessed according to Blois [17], with a slight modification. Different concentrations of the MpEO and silymarin (5–100 μg/ml) were mixed with 1 ml of 0.1 mM DPPH in ethanol solution and 450 μL of 50 mM Tris-HCl buffer (pH 7.4) was added. The solution was incubated at 37 °C for 30 min and the reduction of DPPH free radicals was measured by reading the absorbance at ʎ = 517 nm. Silymarin was used as reference standard. The activity is given as % DPPH scavenging and calculated according to the following equation:

$$ \%\mathrm{DPPH}\  \mathrm{scavenging}=\left[\left(\mathrm{control}\ \mathrm{OD}-\mathrm{sample}\ \mathrm{OD}\right)/\mathrm{control}\ \mathrm{OD}\right]\times 100 $$

The antioxidant activity of MpEO is expressed as IC50, defined as the concentration of MpEO required to cause a 50% decrease in initial DPPH concentration. Each sample was analyzed six times.

### Scavenging of superoxide anion

The influence of MpEO on the generation of superoxide anion was measured according to the method described by Yen & Chen, 1995 [18]. Superoxide anion was generated in a non-enzymatic system and determined by spectrophotometric measurement for the reduction of nitroblue tetrazolium. The reaction mixture, which contained 100 μL of essential oil in ethanol, 800 μL of 1 M phosphate buffer (pH 7.4), 400 μL of distilled water, 100 μL of 0.1 M Na_4_EDTA, 100 μL of 1.5 mM NBT and 50 μL of 0.12 mM riboflavin was incubated at ambient temperature for 5 min, and the color was read at ʎ = 560 nm against blank samples.

$$ \%\mathrm{superoxide}\  \mathrm{anion}\  \mathrm{scavenging}=\left[\left(\mathrm{blank}\ \mathrm{OD}-\mathrm{sample}\ \mathrm{OD}\right)/\mathrm{OD}\ \mathrm{blank}\right]\times 100 $$

Where blank OD is the absorbance of the control reaction and sample OD is the absorbance in the presence of MpEO. The IC50 was calculated from the plot of the inhibition percentage against the essential oil concentration. Each sample was analyzed six times.

### In vivo antioxidant properties

#### Animal

Male *Wistar* rats, weighing about 200–220 g, were purchased from the Central Pharmacy of Tunisia (SIPHAT, Tunisia). They were housed at 22 ± 3 °C with light/dark periods of 12 h and a minimum relative humidity of 40%. The animals had free access to commercial pellet diet (SICO, Sfax, Tunisia) and water ad libitum. The general guidelines for the use and care of living animals in scientific investigations were followed [19]. The handling of the animals was approved by the Tunisian Ethical Committee for the Care and Use of laboratory animals.

#### Experimental design

After acclimatizing to the laboratory conditions for 1 week, 70 rats were divided into 7 groups of 10 animals and treated for 7 days as follow [20]:

The rats of group 1 served as normal control and received saline orally daily for 7 days and were injected with 1 ml/kg BW of just olive oil (the solvent of CCl_4_) on the 7 day. The rats of group 2 served as CCl_4_-hepato and renotoxicity control and were received saline orally daily for 7 days and were injected with 1 ml/kg BW of CCl_4_ and olive oil mixture on the 7 day (a single intraperitoneal injection). The CCl_4_ dose was selected according to the reference dose for chronic oral exposure (RFD) as recommended for CCl_4_ (CASRN 56–23-5) [21].

The rats of group 3 were pretreated orally seven times with a dose of 50 mg/kg BW of reference drug silymarin with an interval of 24 h [22].

The rats of groups 4, 5, 6 and 7 were pretreated orally seven times with doses of 5, 15 and 40 mg MpEO /kg BW, respectively with an interval of 24 h [23].

After pretreatment with either silymarin or MpEO for 7 days, the rats of groups 3, 4, 5 and 6 received a single intraperitoneal injection of CCl_4_ (1 ml/kg BW) on the 7 day.

Rats were killed 24 h after vehicle or CCl_4_ single injection. The animals in the different groups were killed by cervical decapitation to avoid stress conditions.

#### Sample collection

Serum was prepared by centrifugation (1500×*g*, 15 min, 4 °C; Beckman-Coulter, Marseille, France) and stored at −80 °C for further biochemical assays. The liver and kidney tissues were immediately removed and dissected over ice-cold glass slides and a part was homogenized (10% *w*/*v*) with an Ultra Turrax homogenizer in ice-cold, 1.15% KCl-0.01 M sodium, potassium phosphate buffer. Homogenates were centrifuged at 10000×*g* for 20 min at 4 °C. The resulting supernatants were used for immediate lipid peroxidation and protein oxidation determination. Homogenate aliquots were stored at −80 °C for further biochemical assays. Other parts of these livers and kidney tissues were fixed in 10% formaldehyde solution and processed for paraffin sectioning and histological studies.

### Biochemical assays

#### Biochemical markers in plasma

Plasma levels of aspartate aminotransferase (AST), alanine aminotransferase (ALT), alkaline phosphatase (ALP), γ-glutamyl transpeptidase (γ-GT), cholesterol (TC), triglycerides (TG), low-density lipoprotein (LDL), high-density lipoprotein (HDL), creatinine and urea rates were measured in plasma samples by standardized enzymatic procedures using commercial kits from (Biolabo, Maizy, France) on an automatic biochemistry analyzer (Vitalab Flexor E, Diamond Diagnostics, Holliston, MA).

#### Protein quantification

Protein content in liver and kidney tissues were determined according to the method of Lowry et al. [24] using bovine serum albumin as a standard.

#### Lipid peroxidation

Malondialdehyde concentrations (marker for lipid peroxidation) in liver and kidney tissues were determined spectrophotometrically according to Draper & Hadley [25]. Briefly, an aliquot of liver and kidney extracts supernatant was mixed with 1 ml of 5% trichloroacetic acid and centrifuged at 2500×*g* for 10 min. One ml of thiobarbituric acid reagent (0.67%) was added to 500 μl of supernatant and heated at 90 °C for 15 min. The mixture was cooled and the absorbance measured at 532 nm using a spectrophotometer (Jenway UV-6305, Essex, England). The malondialdehyde values were calculated using 1,1,3,3-tetraethoxypropane as standard and expressed as nmol of malondialdehyde/mg of protein.

#### Determination antioxidant enzyme activities in liver and kidney tissue

Catalase (CAT) activity was measured according to Aebi [26]. A total of 20 μL tissue homogenate (about 1.5 mg proteins) was added to 1 ml phosphate buffer (0.1 M, pH 7) containing 100 mM H_2_O_2_. Rate of H_2_O_2_ decomposition was followed by measuring the decrease in absorbance at 240 nm for 1 min. The enzyme activity was calculated using an extinction coefficient of 0.043 mM^−1^ cm^−1^ and expressed in international units (I.U.), i.e. in μmol H_2_O_2_ destroyed/min/ mg protein, at 25 °C.

Superoxide dismutase (SOD) activity was estimated according to Beyer and Fridovich [27]. The reaction mixture contained 50 mM of tissue homogenates in potassium phosphate buffer (pH 7.8), 0.1 mM EDTA, 13 mM L-methionine, 2 mM riboflavin and 75 mM nitro blue tetrazolium (NBT). The developed blue color in the reaction was measured at 560 nm. Units of SOD activity were expressed as the amount of enzyme required to inhibit the reduction of NBT by 50% and the activity was expressed as units/mg of protein, at 25 °C. Glutathione peroxidase (GPx) activity was measured by the procedure of Flohe and Gunzler [28]. One milliliter of reaction mixture containing 0.3 ml of phosphate buffer (0.1 M, pH 7.4), 0.2 ml of 2 mM glutathione (GSH), 0.1 ml of sodium azide (10 mM), 0.1 ml of H_2_O_2_ (1 mM) and 0.3 ml of liver and kidney supernatant were prepared. After incubation at 37 °C for 15 min, the reaction was terminated by adding 0.5 ml 5% TCA. Tubes were centrifuged at 1500×g for 10 min and the supernatant was collected. To 0.1 ml of this reaction supernatant, 0.2 ml of (0.1 M pH 7.4) and 0.7 mL of 5,5 dithiobis-(2-Nitrobenzoic acid) (DTNB, 0.4 mg/ml) were added. After mixing, absorbance was recorded at 420 nm and the enzyme activity was calculated as μmol of GSH oxidized/min/mg protein.

#### Histopathological studies

At the time of sacrifice, the liver and kidney tissues were removed and fixed in 10% formaldehyde solution and washed. The tissues were dehydrated in increasing gradient of ethanol, finally cleared in toluene and embedded in molten paraffin wax. Sections were cut at 4–5 μm thickness and stained with hematoxylin and eosin (H&E). The slides were photographed with an Olympus UTU1X-2 camera connected to an Olympus CX41 microscope (Tokyo, Japan).

The histological damage in liver was quantified by measuring the index of tissue large numbers of inflammatory cells such as lymphocytes together with hepatic sinusoidal inflammation, hepatocyte necrosis and devastating liver architecture. Moreover, the histological damage in kidney was quantified by measuring the index of tissue the glomerular and tubular necrosis. To evaluate the severity of lesions, the degree of liver and kidney damage was graded according to a zero to four-point scoring system [29], where 0 indicates no damage, I indicates slight damage (1–25%), II indicates discrete damage (26–50%), III indicates moderate damage (51–75%) and IV indicates severe damage (76–100%).The tabulation of data and the statistical analysis were made in accordance with the number of animals with established scores. All the parameters were quantified by a single observer who was not aware of the treatment groups.

#### Statistical analysis

All values are expressed as mean ± SE for continues variables or as median with inter quartile range [25%, 75%] where appropriate. The results were analyzed by One-Way Analysis of Variance (ANOVA) followed by Tukey test for multiple comparisons using SPSS for Windows (version. 12) or ANOVA-on-ranks with Dunn’s correction. Differences were considered significant at *p* < 0.05.

## Results

### Chemical constitution of *Mentha piperita* L. leaf essential oil

Chemical composition of MpEO was determined by GC/MS analysis. The compounds, their percentages as well as their retention indices are listed in Table 1. MpEO is a mixture with 26 compounds representing 98.17% of the total oil composition. The most abundant chemicals categories for MpEO are oxygenated monoterpenes (79.50%), followed by monoterpene hydrocarbons (16.23%) and sesquiterpene hydrocarbons (2.44%). The major components of MpEO are menthol (33.59%) and iso-menthone (33.00%). In lower amounts we found a variety of compounds including limonene (8.00%), piperitone (3.20%), 1,8-cineole (2.80%), linalool (2.64%), iso-pulegol (2.40%), caryophyllene (1.95%) and pulegone (1.60%).

**Table 1:** Chemical composition (%) of leaves essential oil from Tunisian *M.piperita* as identified by GC/MS analysis

Peak	Compounds	Retention Time (min)	Percentage (%)
1	α-pinene	4.42	1.80
2	β-pinene	6.07	0.14
3	Sabinene	6.42	0.25
4	Myrcene	7.81	1.30
5	Limonene	8.30	8.0
6	1,8-cineole	8.48	2.80
7	3-octanone	10.13	0.45
8	3-octanol	10.41	0.53
9	Limonene oxide	13.44	0.59
10	α-terpineol	18.62	0.37
11	Linalool	15.73	2.64
12	iso-menthone	15.14	33.00
13	Menthyl acetate	16.35	0.68
14	Iso-pulegol	16.71	2.40
15	Isomenthol	17.29	0.28
16	Neo-iso-menthol	17.90	0.45
17	Menthol	18.25	33.59
18	Pulegone	18.39	1.6
19	Neryl acetate	18.45	0.8
20	Piperitone	19.09	3.2
21	Myrtenol	19.56	0.55
22	Carveol	19.99	0.31
23	Caryophyllene	17.33	1.95
24	Caryophyllene oxide	20.10	0.11
25	Germacrene D	20.80	0.11
26	Δ –Cadinene	21.92	0.27
		Monoterpene hydrocarbons (%)	16.23
		Oxygenated monoterpenes (%)	79.5
		Sesquiterpene hydrocarbons (%)	2.44
		Total (%)	98.17

### Essential oil antioxidant activity

The antioxidant activity of MpEO was compared to that of silymarin, a well-known antioxidant, using two different assays, namely DPPH and superoxide oxygen radicals inhibition, the results are reported in Table 2. DPPH showed for MpEO an IC50 value around 3 times higher than the one recorded for silymarin indicating that antioxidant activity of MpEO was lower than that of silymarin.

**Table 2:** MpEO effects and positive controls on the in vitro free radical(DPPH and superoxide)

	MpEO	Silymarin
50% scavenging concentration (μg/ml) on DPPH radical	61.28 ± 0.02*	21.25 ± 0.13
50% scavenging concentration (μg/ml) on superoxide anion	356.45 ± 0.35*	39.04 ± 1.02

Values are represented as mean ± SEM of six different experiments

*p < 0.05 versus silymarin

### Serum biochemical parameters

The results of biochemical indicators of liver and kidney function are summarized in Tables 3, 4 and 5. The administration of CCl_4_ caused severe hepato and reno-toxicity in the treated rats, as evidenced by the significant elevations of serum ALT, AST, ALP, LDH, γGT, total cholesterol, triglycerides, LDL urea and creatinine levels, while HDL level was decreased compared to control animals.

**Table 3:** Effects of CCl_4_, MpEO and their combination MpEO/CCl_4_ on hepatic markers in serum of control and experimental rats

Treatment and parameters	AST (U/L)	ALT (U/L)	ALP (U/L)	LDH (U/L)	γ-GT (U/L)
Control	164.82 ± 1.28	65.88 ± 1.44	149.61 ± 2.19	20.81 ± 0.52	3.17 ± 0.39
CCl_4_	524.12 ± 5.11^***^	179.23 ± 3.39^***^	217.47 ± 8.55^***^	40.66 ± 0.81^***^	5.43 ± 0.21^***^
SL/CCl_4_	191.15 ± 2.14^###^	87.82 ± 7.61^###^	157.50 ± 5.12^##^	28.62 ± 0.62^###^	3.47 ± 0.06^###^
MpEO^a^/CCl_4_	479.89 ± 36.41	162.02 ± 9.18	202.42 ± 7.62	37.75 ± 1.35	4.45 ± 0.25
MpEO^b^/CCl_4_	356.82 ± 35.77^##^	147.35 ± 5.67^##^	186.74 ± 3.98^#^	34.16 ± 1.35^##^	4.06 ± 0.17^##^
MpEO^c^/CCl_4_	216.80 ± 7.26^###^	97.82 ± 6.98^###^	165.96 ± 5.12^###^	31.64 ± 5.12^###^	3.51 ± 0.20^###^
MpEO^c^	160.80 ± 2.47	63.08 ± 1.16	139.77 ± 7.27	19.89 ± 0.18	2.81 ± 0.24

*AST* aspartate aminotransferase, *ALT* alanine aminotransferase, *ALP* alkaline phosphatase, *LDH* lacatate dehydrogenase and *γGT* gamma glutamyl transferase.^a^MpEO(5 mg/kg BW), ^b^MpEO(15 mg/kg BW), ^c^MpEO(40 mg/kg BW), SL: Silymarin (50 mg/kg BW)

Values are mean ± SEM for eight rats in each group. CCl_4_, MpEO and MpEO/CCl_4_ treated groups vs control group; ** *p* < 0.01, *** *p* < 0.001, CCl_4_ group vs MpEO/CCl_4_ group; ^#^ *p* < 0.05, ^# #^ *p* < 0.01, ^# # #^ *p* < 0.001

**Table 4:** Effects of CCl_4_,MpEO and their combination MpEO/CCl_4_ on lipid profile in serum of control and experimental rats

Treatment and parameters	T-Cholesterol (mmol/l)	T-Triglycerides (mmol/l)	HDL (mmol/L)	LDL (mmol/l)
Control	1.133 ± 0.033	0.736 ± 0.075	1.413 ± 0.023	0.123 ± 0.014
CCl_4_	1.750 ± 0.028^***^	1.743 ± 0.078^***^	0.446 ± 0.074^***^	0.726 ± 0.053^***^
SL/CCl_4_	0.966 ± 0.033^###^	1.043 ± 0.012^###^	1.240 ± 0.030^###^	0.176 ± 0.008^###^
MpEO^a^/CCl_4_	1.606 ± 0.063	1.610 ± 0.041	0.690 ± 0.135	0.640 ± 0.066
MpEO^b^/CCl_4_	1.376 ± 0.076^##^	1.196 ± 0.039^##^	0.873 ± 0.053^##^	0.253 ± 0.024^##^
MpEO^c^/CCl_4_	1.033 ± 0.033^###^	1.050 ± 0.010^###^	1.296 ± 0.039^###^	0.186 ± 0.017^###^
MpEO^c^	1.100 ± 0.115	0.763 ± 0.069	1.210 ± 0.106	0.113 ± 0.006

*TC* Total cholesterol (mmol/l), *HDL* high density lipoprotein (mmol/l), *TG* triglyceride (mmol/l) and *LDL* low density lipoprotein (mmol/l). ^a^MpEO (5 mg/kg BW), ^b^MpEO (15 mg/kg BW), ^c^MpEO (40 mg/kg BW), SL: Silymarin (50 mg/kg BW)

Values are mean ± SEM for eight rats in each group. CCl4, MpEO and MpEO/CCl_4_ treated groups vs control group; ** *p* < 0.01, *** *p* < 0.001, CCl_4_ group vs MpEO/CCl_4_ group; ^#^ *p* < 0.05, ^# #^ *p* < 0.01, ^# # #^ *p* < 0.001

**Table 5:** Effects of CCl_4_, MpEO and their combination MpEO/CCl_4_ on kidney markers in serum of control and experimental rats

Treatment	Urea (mmol/l)	Creatinine (μmol /l)
Control	7.53 ± 0.59	11.12 ± 0.30
CCl_4_	15.41 ± 1.57**	12.79 ± 0.15**
SL/CCl_4_	8.04 ± 0.32^# #^	11.19 ± 0.27^# #^
MpEO^a^/CCl_4_	12.52 ± 0.32	11.94 ± 0.47
MpEO^b^/CCl_4_	10.50 ± 0.29^#^	11.45 ± 0.40^#^
MpEO^c^/CCl_4_	9.44 ± 0.67^# #^	11.39 ± 0.30^# #^
MpEO^c^	7.34 ± 0.38	11.27 ± 0.35

^a^MpEO(5 mg/kg BW), ^b^MpEO(15 mg/kg BW), ^c^MpEO(40 mg/kg BW), SL: Silymarin (50 mg/kg BW)

Values are mean ± SEM for ten rats in each group. CCl4, MpEO and MpEO/CCl_4_ treated groups vs control group; ** *p* < 0.01, *** *p* < 0.001, CCl_4_ group vs MpEO/CCl_4_group; ^#^ *p* < 0.05, ^# #^ *p* < 0.01, ^# # #^ *p* < 0.001

Pretreatment with the MpEO at doses of 15 or 40 mg/kg significantly reduced levels of ALT, AST, ALP, LDH, γGT, total cholesterol, triglycerides, LDL urea and creatinine and increased the level of HDL compared to the CCl_4_ group. It is worth noting that the treatment with 5 mg/kg MpEO did not induce any significant changes in the biochemical parameters (ALT, AST, ALP, LDH, γGT, total cholesterol, triglycerides, LDL, urea, creatinine or HDL) when compared to the CCl_4_ group. Treatment of rats with only MpEO (40 mg/kg BW) did not result in significant alterations in biochemical parameters compared to control rats.

Pretreatment with silymarin (50 mg/kg), used as positive control, significantly decreased the elevated levels of ALT, AST, ALP, LDH, γGT, total cholesterol, triglycerides, LDL urea and creatinine and increased of HDL level as compared to CCl_4_ group. Its effect was comparable in reducing of liver and kidney damage induced by CCl_4_ with that observed for the highest dose of MpEO (40 mg/kg).

### Effects on lipid peroxidation

**Fig. 1:**
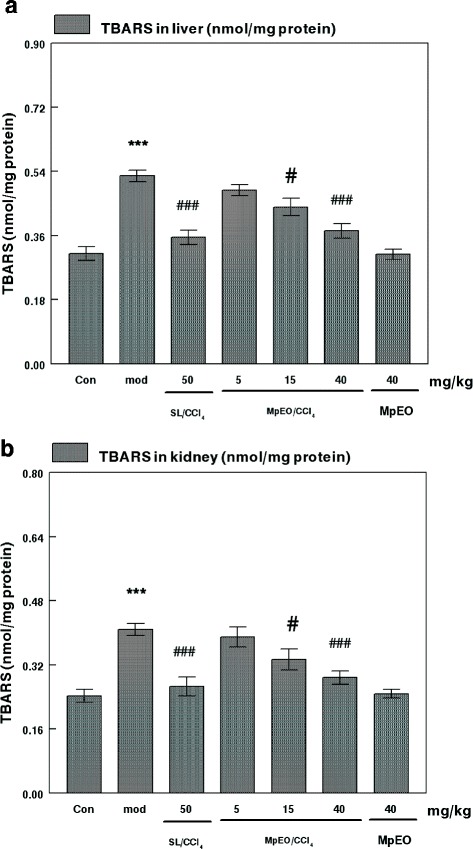
**a** Effects of CCl_4_, MpEO and their combinations MpEO/CCl_4_ on hepatic TBARS of control (Con) and experimental rats. Con, control group; mod, CCl_4_-model group_;_ SL/CCl_4_, silymarin 50 mg/kg + CCl_4_; MpEO/CCl_4_ 5 mg/kg + CCl_4_ group; MpEO/CCl_4_ 15 mg/kg + CCl_4_ group; MpEO/CCl_4_ 40 mg/kg + CCl_4_ group; MpEO 40 mg/kg group. Values are mean ± SEM for ten rats in each group. CCl_4_, MpEO, MpEO/CCl_4_ treated groups vs control group; *p < 0.05, **p < 0.01, *** *p* < 0.001, CCl_4_ group vs (MpEO/CCl_4_) group; #*p* < 0.05, ##*p* < 0.01, ###*p* < 0.001. **b**. Effects of CCl_4_, MpEO and their combinations (MpEO/CCl_4_) on kidney TBARS of control (Con) and experimental rats. Con, control group; mod, CCl4-model group_;_ SL/CCl_4_, silymarin 50 mg/kg + CCl_4_; MpEO/CCl_4_ 5 mg/kg + CCl_4_ group; MpEO/CCl_4_ 15 mg/kg + CCl4 group; MpEO/CCl_4_ 40 mg/kg + CCl4 group; MpEO 40 mg/kg. Values are mean ± SEM for ten rats in each group. CCl_4_, MpEO, MpEO/CCl_4_ treated groups vs control group; *p < 0.05, **p < 0.01, *** *p* < 0.001, CCl_4_ group vs (MpEO/CCl_4_) group; #*p* < 0.05, ##*p* < 0.01, ###*p* < 0.001

TBARS level is widely used as a marker for free radical mediated lipid peroxidation injury. We determined TBARS levels in the liver and kidney tissues of the investigated animals and our results are shown in Fig. 1a, b. The levels of TBARS were significantly increased in both liver and kidney tissues of CCl_4_-treated animals when compared to control untreated rats.

Pre-treatment with the MpEO at doses of 15 and 40 mg/kg BW significantly reduced levels of TBARS in liver and kidney tissues as compared to CCl_4_ group. There was a dose effect; treatment with MpEO at 5 mg/kg BW did not induce any significant decrease in the levels of TBARS in liver and kidney as compared to CCl_4_ group. When rats were treated with only MpEO (40 mg/kg BW), no significant differences in the TBARS values was observed compared to control rat. Pretreatment with silymarin (50 mg/kg) significantly decreased the elevated levels of TBARS in both liver and kidney compared to CCl_4_ control. Moreover, the effect of silymarin (50 mg/kg) in attenuation of TBARS levels in liver and kidney was comparable with highest dose of MpEO (40 mg/kg).

### Effects on antioxidant enzymes

Results presented in Tables 6 and 7 showed a significant decrease in the levels of CAT, SOD, GPx in liver and kidney in CCl_4_-treated group when compared to control group. The decrease in hepatic and kidney CAT, SOD and GPx levels induced by CCl_4_ injection were significantly restored (elevated) in the MpEO and silymarin groups, and this effect was more pronounced with the increase of essential oil concentration. Pretreatment with MpEO at doses of 15 and 40 mg/kg significantly increased levels of hepatic and kidney CAT, SOD and GPx as compared to CCl_4_ group. It is worth noting that the treatment with MpEO at a dose 5 mg/kg did not induce any significant increase in the levels of hepatic and kidney CAT, SOD and GPx as compared to CCl_4_ group. No significant differences in the values were observed in rats treated with MpEO only (40 mg/kg) compared to control rat values. Moreover, the effect of silymarin (50 mg/kg) was comparable in attenuation of levels of hepatic and kidney CAT, SOD and GPx with highest dose of MpEO (40 mg/kg).

**Table 6:** Effects of CCl_4_, MpEO and their combination MpEO/CCl_4_ on the activities of enzymatic antioxidants in liver of control and experimental rats

Treatment	SOD (Units/mg protein)	CAT (μmol H_2_O_2_/min/mg protein)	GPx (μmol GSH/min/mg protein)
Control	16.04 ± 0.11	14.03 ± 0.29	7.66 ± 0.51
CCl_4_	13.60 ± 0.50^***^	11.13 ± 0.37^***^	5.25 ± 0.25^**^
SL/CCl_4_	15.80 ± 0.50^##^	13.48 ± 0.25^###^	7.41 ± 0.16^###^
MpEO^a^/CCl_4_	13.92 ± 0.40	11.34 ± 0.41	5.41 ± 0.31
MpEO^b^/CCl_4_	14.98 ± 0.19^#^	12.32 ± 0.32^#^	6.04 ± 0.17^#^
MpEO^c^/CCl_4_	15.65 ± 0.44^##^	13.20 ± 0.19^###^	7.26 ± 0.22^###^
MpEO^c^	15.83 ± 0.31	13.85 ± 0.33	7.45 ± 0.33

^a^MpEO(5 mg/kg BW), ^b^MpEO (15 mg/kg BW), ^c^MpEO(40 mg/kg BW), SL: Silymarin (50 mg/kg BW)

Values are mean ± SEM for ten rats in each group. CCl4, MpEO and MpEO/CCl_4_ treated groups vs control group; ** *p* < 0.01, *** *p* < 0.001, CCl_4_ group vs MpEO/CCl_4_ group; ^#^ *p* < 0.05, ^# #^ *p* < 0.01, ^# # #^ *p* < 0.001

**Table 7:** Effects of CCl_4_, MpEO and their combination MpEO/CCl_4_ on the activities of enzymatic antioxidants in kidney of control and experimental rats

Treatment	SOD (Units/mg protein)	CAT (μmol H_2_O_2_/min/mg protein)	GPx (μmol GSH/min/mg protein)
Control	15.13 ± 0.22	12.90 ± 0.15	5.68 ± 0.27
CCl_4_	12.12 ± 0.50^***^	10.05 ± 0.34^***^	4.06 ± 0.28^**^
SL/CCl_4_	14.52 ± 0.35^##^	12.23 ± 0.24^###^	5.56 ± 0.14^##^
MpEO^a^/CCl_4_	12.92 ± 0.20	10.80 ± 0.22	4.46 ± 0.33
MpEO^b^/CCl_4_	13.69 ± 0.10^#^	11.02 ± 0.22^#^	5.10 ± 0.28^#^
MpEO^c^/CCl_4_	14.20 ± 0.35^##^	12.04 ± 0.19^###^	5.32 ± 0.06^##^
MpEO^c^	14.91 ± 0.21	12.62 ± 0.33	5.59 ± 0.27

^a^MpEO(5 mg/kg BW), ^b^MpEO(15 mg/kg BW), ^c^MpEO(40 mg/kg BW), SL: Silymarin (50 mg/kg BW)

Values are mean ± SEM for ten rats in each group. CCl4, MpEO and MpEO/CCl_4_ treated groups vs control group; ** *p* < 0.01, *** *p* < 0.001, CCl_4_ group vs MpEO/CCl_4_ group; ^#^ *p* < 0.05, ^# #^ *p* < 0.01, ^# # #^ *p* < 0.001

### Histopathological findings

**Fig. 2:**
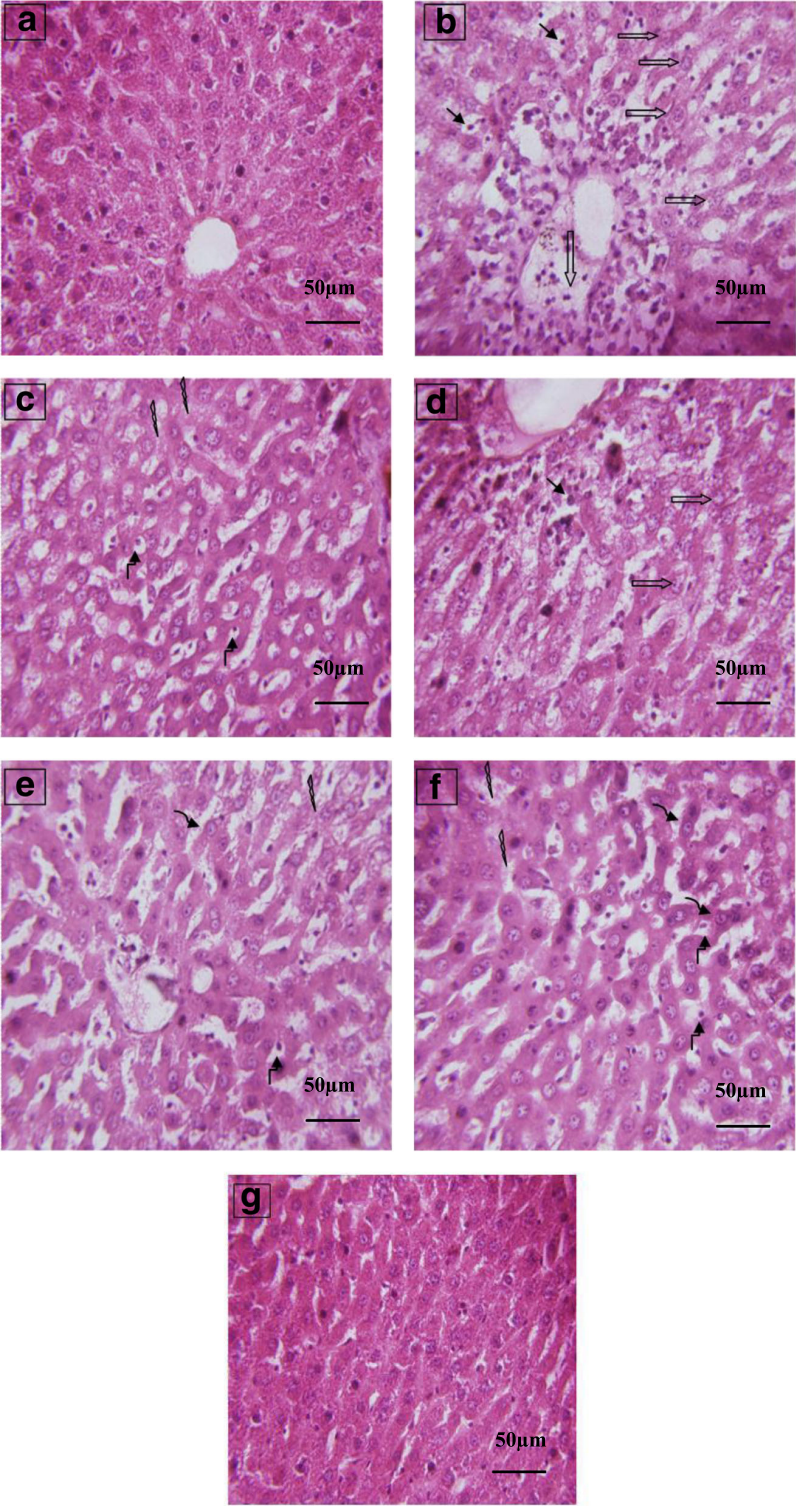
Effect of MpEO on CCl_4_-induced liver damage. **a** Control group; (**b**) CCl_4_-model group showing marked inflammatory cells, necrosis and reduced lesions of necrosis; (**c**) Silymarin 50 mg/kg + CCl_4_ group; (**d**) MpEO 5 mg/kg + CCl_4_ group (**e**); MpEO 15 mg/kg + CCl_4_ group; (**f**) MpEO 40 mg/kg + CCl_4_ group; (**g**) MpEO 40 mg/kg group. Hematoxylin/eosin staining; magnification ×400.  : Marked inflammatory cells;  : Necrosis cells;  : Reduced lesions of necrosis;  : Regeneration area;  : Prominent nucleolus;  : Mild inflammatory cells

The normal liver architecture was observed in liver histology of control group (Fig. 2a). Large numbers of inflammatory cells such as lymphocytes together with hepatic sinusoidal inflammation, hepatocyte necrosis and devastating liver architecture were observed in the CCl_4_ group (Fig. 2b). However, pretreatment with MpEO (40 mg/kg, Fig. 2f) can remarkably ameliorate the histopathological hepatic lesions induced by administration of CCl_4_. MpEO 15 mg/kg showed very few inflammatory cells along with prominent nucleolus (Fig. 2e). The highest dose of MpEO (40 mg/kg) and silymarin (50 mg/kg) significantly attenuated the damaged liver depicting marked focal regenerative changes which are illustrated by presence of actively dividing cells with a prominent nucleolus (Fig. 2f). In addition, silymarin at the dose of 50 mg/kg has shown to produce hepatoprotection evidenced by area of regeneration and dark nucleus (Fig. 2c). The histological pattern was almost normal in rats treated with MpEO oil alone. By analyzing the histopathological scoring attributed to the liver tissues it is possible to note that the highest dose of MpEO (40 mg/kg) or silymarin (50 mg/kg) pretreatment just conferred good protection on CCl_4_-induced liver damage (Table 8).

**Table 8:** Grades of inflammatory cells and cellular necrosis in rat liver

Pathologic grading of inflammatory cells and cellular necrosis								
Groups	Zero	I	II	III	IV	N	*P* value vs Control	*P* value vs CCl_4_
Control	10	0	0	0	0	10	–	
CCl_4_^***^	0	1	3	3	3	10	*P* < 0.001	–
SL/CCl_4_^###^	8	2	0	0	0	10	ns	*P* < 0.001
MpEO^a^/CCl_4_	0	2	6	1	1	10	*P* < 0.05	ns
MpEO^b^/CCl_4_^#^	6	4	0	0	0	10	ns	*P* < 0.05
MpEO^c^/CCl_4_^###^	7	3	0	0	0	10	ns	*P* < 0.001
MpEO^c^	9	1	0	0	0	10	ns	*P* < 0.001

^a^MpEO (5 mg/kg BW), ^b^MpEO (15 mg/kg BW), ^c^MpEO (40 mg/kg BW), SL: Silymarin (50 mg/kg BW)

**Fig. 3:**
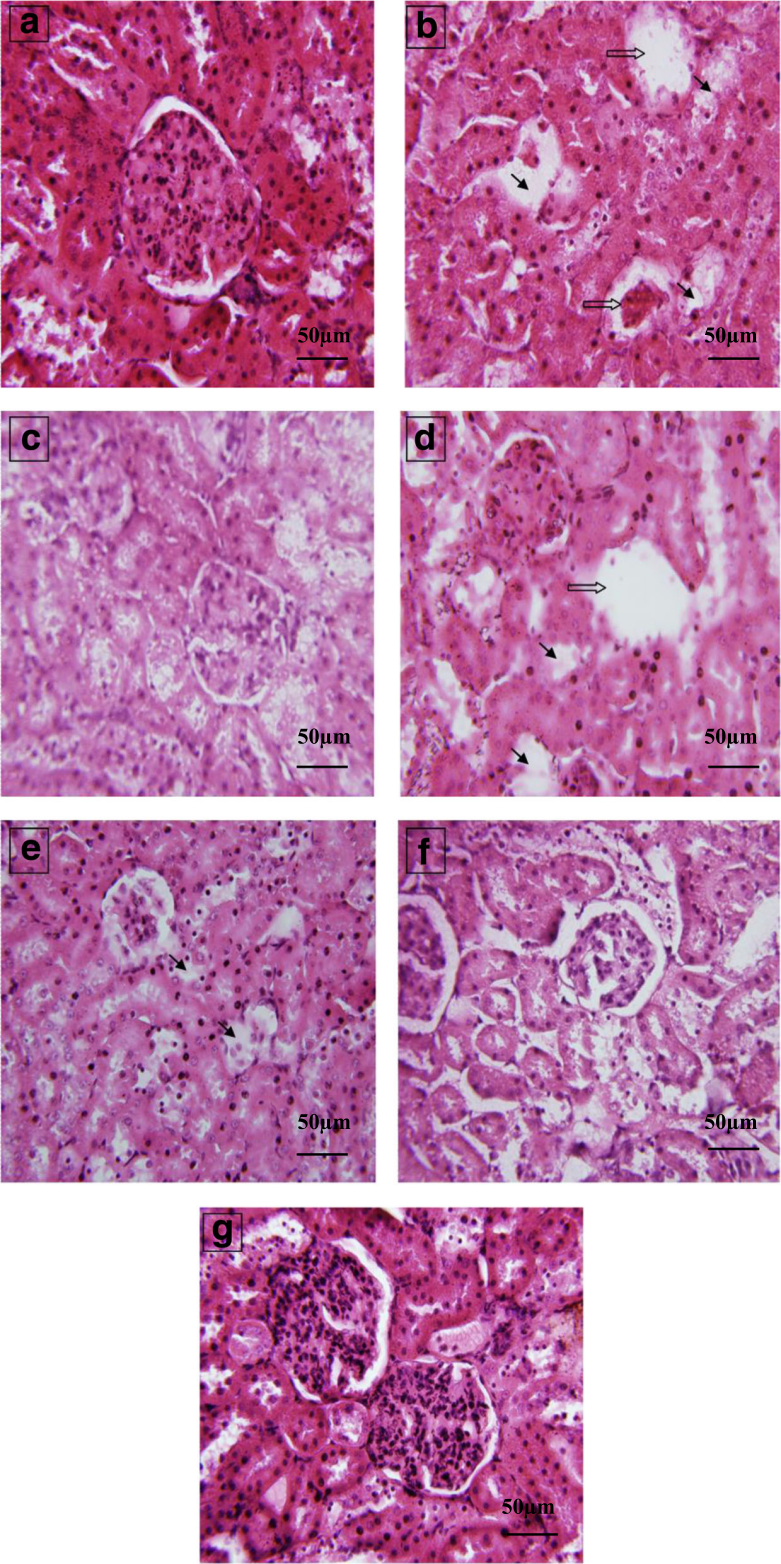
Effect of MpEO on CCl_4_-induced kidney damage. **a** Control group; (**b**) CCl_4_-model group showing some nephrotoxic lesions, as evidenced by the glomerular and tubular necrosis; (**c**) Silymarin 50 mg/kg + CCl_4_ group; (**d**) MpEO 5 mg/kg + CCl_4_ group (**e**); MpEO 15 mg/kg + CCl_4_ group; (**f**) MpEO 40 mg/kg + CCl_4_ group; (**g**) MpEO 40 mg/kg group. Hematoxylin/eosin staining; magnification ×400.  : glomerular necrosis;  : necrosis in epithelial cells of the proximal tubules

Kidney sections of normal histological appearance (Fig. 3a) and the CCl_4_ control group showed some nephrotoxic lesions, as evidenced by the glomerular and tubular necrosis (Fig. 3b). However, pretreatment with MpEO (40 mg/kg, Fig. 3f) can remarkably ameliorate the histopathological kidney lesions induced by administration of CCl_4_ (Fig. 3f). In addition, silymarin at the dose of 50 mg/kg has shown to produce renoprotection evidenced by amelioration the histopathological kidney lesions induced by injection of CCl_4_ (Fig. 3c). The histological pattern in kidney was almost normal in rats treated with MpEO alone. By analyzing the histopathological scoring attributed to the kidney tissues it is possible to note that the highest dose of MpEO (40 mg/kg) or silymarin (50 mg/kg) pretreatment just conferred good protection on CCl_4_-induced kidney damage (Table 9).

**Table 9:** Grades of glomerular and epithelial cells of the proximal tubules necrosis in rat kidney

Pathologic grading of glomerular and epithelial cells of the proximal tubules necrosis								
Groups	Zero	I	II	III	IV	N	*P* value vs Control	*P* value vs CCl_4_
Control	10	0	0	0	0	10	–	
CCl_4_^***^	0	2	2	3	3	10	*P* < 0.001	–
SL/CCl_4_^###^	8	2	0	0	0	10	ns	*P* < 0.001
MpEO^a^/CCl_4_	0	2	5	3	0	10	*P* < 0.05	ns
MpEO^b^/CCl_4_^#^	6	4	0	0	0	10	ns	*P* < 0.05
MpEO^c^/CCl_4_^###^	7	3	0	0	0	10	ns	*P* < 0.001
MpEO^c^	9	1	0	0	0	10	ns	*P* < 0.001

^a^MpEO (5 mg/kg BW), ^b^MpEO (15 mg/kg BW), ^c^MpEO (40 mg/kg BW), SL: Silymarin (50 mg/kg BW)

## Discussion

Chemical composition of the essential oil obtained from MpEO was determined by GC-MS analysis. The compounds, their percentages as well as the retention indices are listed in Table 1. The essential oil is a complex mixture with 26 compounds representing 98.17% of the total oil composition. The major component of the essential oil is menthol (33.59%) followed by iso-menthone (33.00%). In lower amounts we found a variety of compounds including limonene (8.00%), piperitone (3.20%), 1,8-cineole (2.80%), linalool (2.64%), iso-pulegol (2.40%), caryophyllene (1.95%) and pulegone (1.60%). The obtained results are in accordance with previous studies of *M. piperita* oils from Turkey, Spain (Barcelona), Norway and Poland that also had menthone and menthol as their most important components [30–32]. On the other hand, the composition of the essential oil from Iran is totally different, with α-terpinene (19.70%), isomenthone (10.30%), trans-carveol (14.50%), pipertitinone oxide (19.30%) and β-caryophyllene (7.60%) as the major compounds, and also the oil from the Girona region (Spain) is different, where limonene and 1.8-cineole, eucalyptol are the main compounds (33.37% and 30.75%, respectively) [33, 34].

These studies showed variable chemotypes of *M. piperita* L. extracts with various major oil components. Differences in chemical composition observed for essential oils is likely related to abiotic factors such as soil type and climate specific regions of provenance samples and geographical factors [35]. Furthermore, menthol and iso-menthone, found at relatively high concentrations in the MpEO used in the present study, have been reported to exhibit anti-inflammatory activity [14], making MpEO use a promising candidate against oxidative damage of the liver and kidney following an intraperitoneal administration of CCl_4._ To be noted: working with natural extracts, the antioxidant activity is considered to be primary related to the major active compounds in the essential oil such as menthol and its derivatives [15]. However the antioxidant activity could also come from a minor compound interacting in a synergistic or antagonistic way, to create an effective system against free radicals [36, 37], this has to be realized when evaluating different MpEO preparations.

Liver injury after CCl_4_ exposure is characterized by the elevated levels of serum hepatic marker enzymes indicating the cellular leakage and loss of functional integrity of hepatic membrane architecture. High levels of ALT, AST, ALP, LDH and γGT activities are sensitive indicators of liver cell injury and are most helpful in recognizing hepatic diseases [38]. CCl_4_-treated rats show increased activities of these enzymes, reflecting damage to the liver cells or changes in the cell membrane permeability leading to leakage of enzymes from cells to the circulation [39]. In the present study increased levels of serum hepatic markers suggested that an extensive liver injury was occasioned by CCl_4_ due to increased lipid peroxidation which had the ability to cause membrane damage. It is now generally accepted that CCl_4_ hepatotoxicity is the result of reductive dehalogenation, which is catalyzed by its specific isoenzyme of cytochrome P450 2E1, and which forms the highly reactive free radical. Hence, the suppression of P450 2E1 could result in reduced levels of reactive metabolites, and thus decreased tissue damage [40].

The liver plays a fundamental role in the metabolism of lipids. Injection of CCl_4_ caused a significant increase in the triglyceride, total cholesterol, and LDL levels and decrease in HDL level. Increase in the cholesterol levels might be due to the increased esterification of fatty acids, inhibition of fatty acid β-oxidation, and decreased excretion of cellular lipids [41]. CCl_4_ stimulates the transfer of acetate into liver cells (probably by increasing access to acetate) and leads to an increase in cholesterol synthesis. It also increases the synthesis of fatty acids and triglyceride from acetate and enhances lipid esterification [42]. The accumulation of triglyceride in liver might occur due to the inhibition of lysosomal lipase activity and VLDL secretion [43].

The administration of CCl_4_ induced also renal toxicity evidenced by an elevation of serum creatinine and urea [44, 45]. These pathological changes can also be attributed to damages touching the structural integrity of nephrons [46], which is consistent with reports confirming that the level of serum creatinine increases only if at least half of the kidney nephrons are already damaged [47].

Treatment of rats with MpEO prior to CCl_4_ exposure resulted in a dramatically protective effect against acute hepato and renotoxicity and oxidative stress, which was further also confirmed by the hepatic histopathological examinations. The stimulation of hepatic regeneration makes the liver more resistant to damage by the toxin [48]. Treatment with silymarin (50 mg/kg) or MpEO (40 mg/kg) significantly decreased the elevated levels of ALT, AST, ALP, LDH, γGT, total cholesterol, triglycerides, LDL urea and creatinine and increased of HDL level as compared to CCl_4_ group. Pharmacological studies have shown that essential oil derived from various plant materials possesses anti-inflammatory activities [49, 50] Knowing that sesquiterpenes have excellent anti-inflammatory activities [51], the anti-inflammatory activity of *M. piperita* L. leaf essential oil could be partly explained by the presence of sesquiterpenes, such as spathulenol, cadinene, caryophyllene and caryophyllene oxide. The ethanolic extract of parsley leaves also showed significant anti-inflammatory [52] and antioxidant activities [53, 54] which may contribute to its hepatoprotective action. Furthermore, menthol and iso-menthone, found at relatively high concentrations in the MpEO used in the present study, have been reported to exhibit anti-inflammatory activity [14].

In the present study, the two fold increase in the TBARS levels and reduce activity of SOD, CAT and GPx observed in liver and kidney homogenate of CCl_4_-intoxicated rats. Silymarin significantly reversed CCl_4_-induced TBARS levels elevation but values obtained with MpEO at the highest dose was comparable in attenuation of TBARS levels in liver and kidney. Silymarin reversed CCl_4_-induced SOD, CAT and GPx activities decrease but values obtained with MpEO at the highest dose (40 mg/kg) was comparable in attenuation of SOD, CAT and GPx activities in liver and kidney.

These results suggested that MpEO could exert its antioxidant and/or radical scavenging activities thus preventing the formation of the carbon free radicals originated from CCl_4_ metabolism as well as ROS and peroxidation products. This hypothesis is supported by the recent findings on the in vitro antiradical and antioxidative activities of MpEO [16]. Previous studies showed that menthol and its derivatives were the major compounds responsible for antioxidant activity of MpEO [15].

In the present study, the rats of group 2 served as CCl_4_-hepato and renotoxicity control and rats of groups 3, 4, 5 and 6 were injected with 1 ml/kg BW of CCl_4_ and olive oil mixture on day seven (a single intraperitoneal injection). It has already been shown that a single dose of CCl_4_ initiates lipid peroxidation [55–58] that results in the disruption of cellular and organelle membrane integrity and subsequent leakage of cellular contents into the blood [59, 60]. CCl_4_ is further well known to induce fibrosis of the hepatic tissue that may further progress to cirrhosis if the stimuli persists [61–65]. Thus, a single CCl_4_ injection in mice can be used as an attractive and highly reproducible model of liver regeneration after toxic injury. The first appearance of histological fibrosis and scarring fibers is usually observed after repeated CCl_4_ treatment for 2 to 3 weeks, depending on the dosage and mouse strains used [66]. In the present work, the hepatic histoarchitecture of the CCl_4_-treated rats resulted large numbers of inflammatory cells such as lymphocytes along with hepatic sinusoidal inflammation, hepatocyte necrosis and devastating liver architecture The highest dose of MpEO (40 mg/kg) or silymarin (50 mg/kg) significantly attenuated the damaged liver depicting marked focal regenerative changes which are illustrated by presence of actively dividing cells with a prominent nucleolus. The administration of MpEO reducing the histological alterations in liver provoked by CCl_4_ was quite noticeable. In fact, the histological changes seen in the kidney of rats treated with CCl_4_ were characterized by some nephrotoxic lesions, as evidenced by the glomerular and tubular necrosis. Our results confirmed previous findings of Ozturk et al. [67] who had found degenerative changes in kidney of rats exposed to CCl_4_. The results suggest that MpEO treatment prior to CCl_4_ intoxication could prevent the CCl_4_-induced alterations in kidney tissues of treated animals.

## Conclusions

The contents of MpEO not only protect the integrity of plasma membrane but, at the same time, increased the regenerative and reparative capacity of the liver and kidney. These results suggest that the compound present in MpEO has hepatorenal protective effects against CCl_4_ induced oxidative stress in rats. Further investigations are essential to elucidate the precise mechanism of active agents of MpEO protection against CCl_4_-induced hepatotoxicity and nephrotoxicity and it has to be tested against other biological parameters.

## Abbreviations

BHT: Butylated hydroxytoluene
CAT: Catalase
DPPH: 1-Diphenyl-2-picrylhydrazyl
DTNB: 5,5′-Dithio-bis(2-nitrobenzoic acid)
EDTA: Ethylenediaminetetraacetic acid
GPX: Glutathione peroxidase
GSH: Glutathione
*M. piperita*: *Mentha piperita*
MpEO: *Mentha piperita* essential oil
NBT: Nitroblue Tetrazolium
ROS: Reactive oxygen species
SD: Standard deviation
SOD: Superoxide dismutase
TBA: Thiobarbituric Acid
TBARS: Thiobarbituric acid reactive substances
TBS: Tris Buffered Saline
TCA: Trichloroacetic Acid
TRIS: Trishydroxymethyl aminomethane
